# Hypocotyl acupuncture: an efficient and practical protocol for transformation and genome editing in *Salvia miltiorrhiza*

**DOI:** 10.1111/tpj.71103

**Published:** 2026-10-07

**Authors:** Chaoyang Wang, Jiangbin Liu, Chang Liu, Cong Liu, Jiaqi Cui, Jianhua Wang, Xiangui Mei, Zhenqiao Song, Xingfeng Li

**Affiliations:** ^1^ Agronomy College Shandong Agricultural University Tai'an 271018 China; ^2^ State Key Laboratory of Wheat Improvement Shandong Agricultural University Tai'an 271018 China

**Keywords:** technical advance, *Salvia miltiorrhiza*, *In planta* transformation, medicinal plants, tissue culture‐free

## Abstract

*Salvia miltiorrhiza* Bunge is a valuable medicinal plant, yet its genetic manipulation has been limited by the lack of efficient and practical transformation methods. Here, we report a simplified, tissue culture‐independent strategy enabling Agrobacterium‐mediated gene delivery under non‐sterile conditions, termed hypocotyl acupuncture. The method employs needle‐assisted inoculation of intact seedlings at the hypocotyl, completely bypassing callus induction, regeneration, and repeated subculture. Following inoculation, EGFP‐positive hairy roots are selectively cultivated for approximately 4 months and then induced to regenerate shoots within one additional month, yielding transgenic plantlets in approximately 5 months. Systematic optimization achieved a transformation efficiency of approximately 55% (defined as the percentage of explants producing EGFP‐positive regenerants). ACCC10060 was selected as the optimal strain based on efficiency and root morphology. CRISPR/Cas9‐mediated genome editing generated mutation frequencies of 53.8%–88.9% with no off‐target effects. Targeted mutagenesis of *SmCPS1*, a gene encoding a key rate‐limiting enzyme in tanshinone biosynthesis, resulted in altered root pigmentation. The method was successfully extended to *Astragalus membranaceus*, *Platycodon grandiflorus*, and *Isatis indigotica*, achieving transformation efficiencies of 25%–55%. This study provides an efficient, practical, and broadly applicable tool for genome editing and functional genomics in medicinal plants.

## Significance Statement

Despite its pharmaceutical importance, genetic manipulation of *Salvia miltiorrhiza* remains constrained by the limited efficiency and practicality of existing tissue culture‐free transformation approaches. We show that hypocotyl acupuncture enables transformation efficiencies of up to 55% and supports genome editing in *S. miltiorrhiza* without callus induction or aseptic procedures, and that this strategy can be extended to three additional medicinal plant species, providing a practical platform for functional genomics and genome engineering.

## INTRODUCTION

The root of *Salvia miltiorrhiza* (Danshen) is widely used in the treatment of cardiovascular and cerebrovascular diseases and represents one of the most important traditional Chinese medicinal herbs. It exhibits a broad spectrum of pharmacological activities, including blood circulation promotion, blood stasis elimination, and antitumor, antibacterial, anti‐inflammatory, anti‐allergic, and anti‐lipid peroxidation effects (Li et al., 2018; Shi et al., 2024). Due to its important medicinal value, extensive clinical application, compact genome size, and increasing availability of genomic resources, *Salvia miltiorrhiza* has emerged as an important model system for medicinal plant research (Pan et al., 2023; Song et al., 2020; Xu et al., 2016). However, the tanshinone biosynthesis pathway remains incompletely characterized. Furthermore, conventional transformation methods have limited the throughput and speed of genetic manipulation in *S. miltiorrhiza*, and the overall transformation process remains substantially slower than that achieved in model plants such as *Arabidopsis thaliana* and *Nicotiana tabacum*.

Substantial progress has recently been made in genetic transformation and genome editing of *S. miltiorrhiza*. The Cut‐Dip‐Budding (CDB) system, a non‐sterile, tissue culture‐independent method, was first established in 2023 and subsequently applied to *S. miltiorrhiza* (Cao et al., 2023, 2024). In parallel, efficient CRISPR/Cas9 protocols (Hsu et al., 2024), base editing systems (Yao et al., 2025), and prime editing platforms (Yao et al., 2026; Yu et al., 2026) have been developed, substantially expanding the precision genome editing toolbox for medicinal plants. Despite these advances, transformation efficiency, scalability, and operational simplicity remain important challenges. Here, we describe a hypocotyl acupuncture method that complements existing approaches by offering a transformation efficiency suitable for batch‐scale application, non‐sterile operation, and ease of handling, and demonstrate its applicability across multiple medicinal plant species.

## RESULTS

We developed a hypocotyl needle puncture procedure for transgenic root induction and subsequent regeneration (Figure 1A). The complete workflow was summarized in Figure 2. *Salvia miltiorrhiza* seeds were germinated for 10 days, yielding seedlings with a well‐developed hypocotyl (Figure 1Aa). The hypocotyl was punctured and infiltrated with an *Agrobacterium* suspension (OD_600_ ≈ 1.0) (Figure 1Ab), briefly soaked (Figure 1Ac), and then transplanted into nutrient‐rich substrate (Figure 1Ad). EGFP‐positive transgenic roots emerged approximately 2 weeks post inoculation. The specificity of EGFP fluorescence was further confirmed by comparison with non‐transformed *S. miltiorrhiza* controls under identical imaging conditions (Figure S1). Root growth and EGFP fluorescence were monitored for up to 4 months, during which a primary root system developed and non‐transgenic roots were removed based on the absence of fluorescence (Figure 1Ae–h,k–n). After 4 months of cultivation, the transgenic root system had reached the target fresh weight threshold (≥3.0 g, Table S1). At this stage, the final transgenic root system was obtained (Figure 1Ai,o). Transgenic root segments were then excised and cultivated for approximately 1 month to induce shoot regeneration (Figure 1Aj,p). The main experimental procedures are summarized in Supplementary Methods (S1–S8).

**Figure 1:**
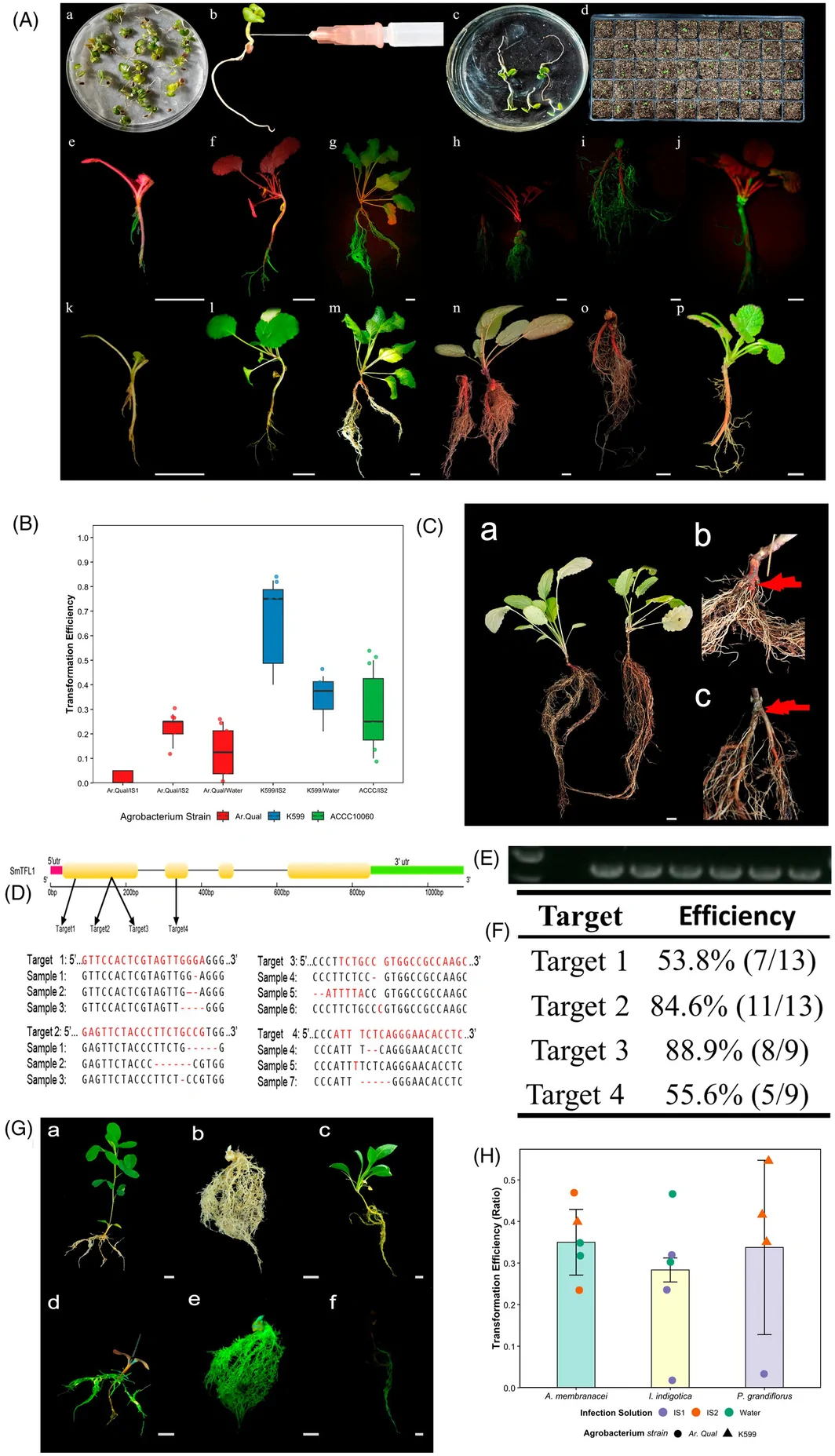
Hypocotyl injection transformation and genome editing in medicinal plants. (A) Schematic flowchart of key steps: a–d, inoculation, soaking, and transplantation of 10‐day‐old seedlings (a, normal seedling; b, hypocotyl injection; c, soaking; d, transplantation); e–j, EGFP fluorescence: e–h, time‐course monitoring (10–15 days, 1 month, 2 months, 4 months) of transgenic roots; i, final root system at 4 months; j, transgenic plantlet; k–p, bright‐field images: k–n, time‐course monitoring (10–15 days, 1 month, 2 months, 4 months) of transgenic roots; o, final root system at 4 months; p, transgenic plantlet. (B) Comparison of transformation efficiency in *S. miltiorrhiza* using different infection solutions and *Agrobacterium* strains. Data are presented as mean ± SD (n = 20). (C) Phenotypic comparison of roots induced by different *Agrobacterium* strains: a, Overall view of roots induced by strains K599 and ACCC10060, b, Abnormal root phenotype induced by K599, c, Normal root phenotype induced by ACCC10060 (red arrows in b and c indicate infection sites). (D) Structure of the target gene for CRISPR/Cas9 editing and sequence alignment. The gene structure diagram shows positions of the four gRNAs; alignment chromatogram shows representative Cas9‐induced mutation patterns. (E) PCR verification of EGFP‐positive transgenic roots: Lane 1, DNA Marker (upper band 750 bp, lower band 500 bp), Lane 2, Negative control, Lanes 3–8, Positive transgenic lines. (F) Analysis of gene editing efficiency (editing efficiencies for the four target sites). (G) Visualization of transgenic root expression in other medicinal plant species: a‐c, Bright‐field morphology of *A. membranaceus*, *P. grandiflorus*, and *I. indigotica*; d‐f, Corresponding EGFP fluorescence in roots. Scale bars: 1 cm. (H) Transformation efficiencies in three additional medicinal species (*A. membranaceus*, *I. indigotica*, and *P. grandiflorus*) under various experimental conditions. Data are presented as mean ± SD.

**Figure 2:**
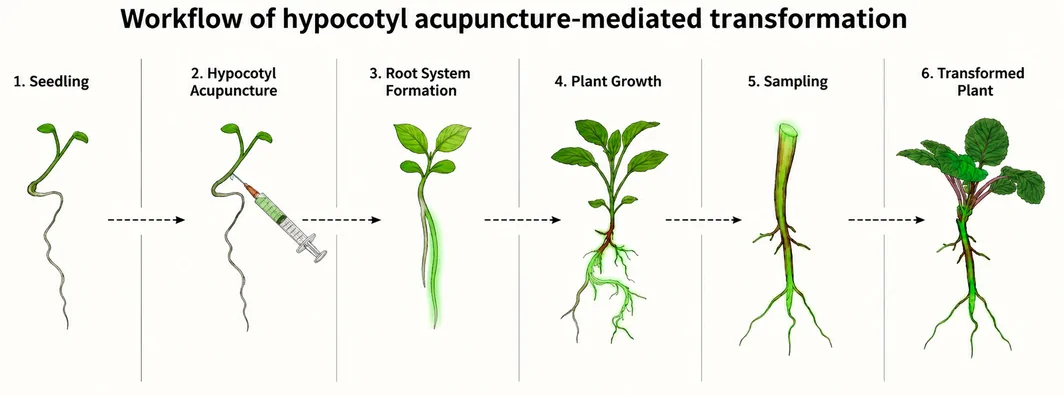
Workflow of hypocotyl acupuncture‐mediated transformation. The diagram illustrates the major steps of the hypocotyl acupuncture procedure, including seedling preparation, hypocotyl acupuncture, root system formation, plant growth, sampling, and generation of transformed plants.

*S. miltiorrhiza* possesses a natural capacity for propagation via root segments (Zhang et al., 2016). To determine the optimal root segment size for efficient shoot regeneration, we evaluated sprouting efficiency across a range of fresh weights. Sprouting efficiency was closely related to root segment fresh weight: segments ≥3.0 g exhibited high sprouting efficiency (76.7–83.3%), with no significant difference between the 3.0 g and 3.5 g groups, while both were significantly higher than those ≤2.0 g (one‐way ANOVA followed by Duncan's multiple range test, *P* < 0.05; Figure S2; Table S1). Based on these results, a threshold of ≥3.0 g was adopted. In this study, transgenic root segments reached this threshold after approximately 4 months of cultivation. Therefore, the entire process from inoculation to shoot regeneration was completed in approximately 5 months.

Transformation efficiency was systematically optimized by evaluating three key factors: Agrobacterium strain, infection solution, and OD_600_ value (Figure 1B). Transformation efficiency was defined as the percentage of inoculated explants yielding EGFP‐positive regenerated plantlets. Type III sum of squares ANOVA (Table S2) revealed that both strain (F = 19.156, *P* = 1.60 × 10^−6^) and infection solution (F = 7.779, *P* = 1.44 × 10^−3^) significantly affected transformation efficiency, whereas OD_600_ value did not (F = 0.13, *P* = 0.7209). Among the infection solutions, Solution 2 gave the best overall performance. For individual strains, K599 achieved a peak transformation efficiency of 85% at an OD_600_ = 1.1 under Solution 2. ACCC10060 reached a peak transformation efficiency of approximately 55% also with Solution 2 at OD_600_ = 0.9. Ar.Qual showed a peak transformation efficiency of 30% under the conditions tested. The survival rate of hypocotyl‐punctured seedlings ranged from 70% to 85%, while the non‐punctured control group showed 90–100% survival (Table S3). These results indicate that the procedure caused limited stress but did not significantly affect seedling viability.

Despite its high transformation efficiency, K599 consistently induced aberrant root morphologies. Roots induced by ACCC10060 and Ar.Qual typically displayed a well‐defined dominant primary root with regularly spaced lateral roots, conforming to normal developmental patterns. In contrast, K599‐mediated transformation consistently resulted in excessive root proliferation at the infection site, producing thin, densely clustered, and entangled roots that were morphologically unsuitable for subsequent cultivation and phenotypic analyses (Figure 1C). Although K599 achieved the highest peak transformation efficiency (85%), the aberrant root architecture severely limited the utility of the resulting material, precluding reliable isolation and long‐term maintenance of individual transgenic root lines. Therefore, by comprehensively evaluating both transformation efficiency and post‐induction root architecture, strain ACCC10060 was selected as the optimal candidate for subsequent experiments.

To evaluate the applicability of this genetic transformation system, we first targeted TFL1 (TERMINAL FLOWER 1), a flowering‐related gene in *S. miltiorrhiza* (Figure 1D, Table S4). Genomic DNA from EGFP‐positive regenerated roots was confirmed by PCR (Figure 1E). The editing efficiency of the CRISPR/Cas9 system was evaluated by next‐generation sequencing (NGS). Across two independent experiments, the mutation frequencies at the four gRNA target sites ranged from 53.8% to 88.9% (Figure 1F).

To further assess editing specificity, all predicted coding sequence (CDS) of *SmTFL1* off‐target sites harboring up to three mismatches relative to the sgRNA were scrutinized in three independent edited lines using targeted Nanopore sequencing. No off‐target mutations were detected at any of the examined loci (Table S5), demonstrating the high targeting fidelity of the CRISPR/Cas9 system in the tested lines.

To further demonstrate the utility of the established genome‐editing system, we targeted *copalyl pyrophosphate synthetase gene 1* (*SmCPS1*), encoding a key rate‐limiting enzyme gene involved in the tanshinone biosynthetic pathway (Cheng et al., 2014; Guo et al., 2016). Roots regenerated from *SmCPS1‐*edited plants displayed a visibly altered pigmentation phenotype, characterized by a lighter orange‐yellow coloration in contrast to the deep red pigmentation characteristic of wild‐type controls (Figure S1). Genotyping of *SmCPS1*‐edited plants by Nanopore sequencing revealed distinct editing patterns: one plant exhibited a mosaic pattern containing multiple mutation events, one showed a homozygous genotype, and the remaining plants displayed heterozygous/biallelic patterns, with wild‐type reads accounting for less than 5% of total reads, indicating stable target‐site mutations (Figure S3; Table S6). HPLC analysis revealed that tanshinone accumulation was markedly reduced in *SmCPS1*‐edited roots, with several major compounds showing undetectable chromatographic peaks compared with wild‐type roots (Figure S4). These results were consistent with the altered root pigmentation and confirmed the feasibility of this platform for functional gene validation.

To further evaluate the robustness of the method, hypocotyl acupuncture has been performed using Baihua Danshen (white‐flowered type) from Laiwu and Zihua Danshen (purple‐flowered type) from Longting, Xintai, both representing common production regions of *S. miltiorrhiza* in Shandong Province. EGFP‐positive hairy roots were observed 2 weeks after inoculation. Transformation frequencies were 45% for Baihua Danshen and 40% for Zihua Danshen (Table S2), supporting the applicability of the method across diverse *S. miltiorrhiza* germplasm.

To evaluate the broader applicability of the optimized hypocotyl injection method, we tested it in three additional medicinal plant species: *Astragalus membranaceus*, *Platycodon grandiflorus*, and *Isatis indigotica*, resulting in successful transformation in all species (Figure 1G,H; Table S7). Transformation efficiency ranged from 25 to 45% in *A. membranaceus*, from 30 to 45% in *I. indigotica* (using Ar.Qual in water), and reached up to 55% in *P. grandiflorus* under specific conditions with K599 (OD_600_ ≈ 1.0, Solution 2). Notably, the root abnormalities associated with K599 in *S. miltiorrhiza* were not apparent in the other species.

## DISCUSSION

In this study, we established a simplified, tissue culture‐independent transformation strategy for *S. miltiorrhiza* that enables *Agrobacterium*‐mediated gene delivery under non‐sterile conditions. By bypassing callus induction, regeneration, and repeated subculture, this approach substantially simplifies the transformation workflow and reduces labor requirements. Transformation is achieved directly on intact seedlings via needle‐assisted infection, allowing plants to be maintained under routine growth conditions throughout the process.

Tissue culture‐independent transformation systems have been previously reported in *S. miltiorrhiza*, including the CDB method (Cao et al., 2023, 2024), which has also been extended to soybean (Cao et al., 2026). In *S. miltiorrhiza*, the CDB method enables direct plant regeneration but exhibits limited efficiency, with a reported transformation efficiency of approximately 6–7% (7 transformants from 109 individuals). In this context, the present study achieves a transformation efficiency of up to approximately 55% in *S. miltiorrhiza*, thereby establishing a scalable and reliable platform for efficient transformation in *S. miltiorrhiza*.

In our study, two target genes were selected to validate the genome‐editing performance of the platform. A flowering‐related gene TFL1 served to quantify editing efficiency (53.8–88.9%). *SmCPS1* provided phenotypic validation: edited roots displayed altered pigmentation consistent with disrupted metabolite accumulation. These results, obtained using a conventional CRISPR/Cas9 system, demonstrate the compatibility of the hypocotyl acupuncture platform with existing genome‐editing tools. Recent advances, including optimized CRISPR/Cas systems (Hsu et al., 2024; Zheng et al., 2025), base editing (Yao et al., 2025), and prime editing (Yao et al., 2026; Yu et al., 2026), have further expanded the toolbox for functional genomics in this species. However, these approaches rely heavily on efficient DNA delivery systems. The hypocotyl acupuncture platform, with its high transformation efficiency and simplified workflow, may serve as a practical delivery solution for these advanced editing technologies, and their integration represents a promising direction for future studies.

The relatively high transformation efficiency observed in this study is likely associated with the use of hypocotyl tissues from young seedlings combined with localized needle‐induced wounding. Compared with mature organs such as leaves or petioles, which contain a higher proportion of differentiated cells, the hypocotyl is a juvenile tissue characterized by low cellular differentiation and high regenerative competence. Such juvenile tissues generally exhibit enhanced susceptibility to *Agrobacterium*‐mediated T‐DNA integration (Gelvin, 2010; Ikeuchi et al., 2013). Mechanical wounding further enhances infection efficiency by improving bacterial access to internal tissues and inducing phenolic compound release, which activates *Agrobacterium* vir genes and promotes T‐DNA transfer (Gelvin, 2003; Pitzschke & Hirt, 2010). Importantly, the effectiveness of wounding depends on injury intensity, and controlled, minimally invasive damage is generally more favorable than extensive tissue disruption, as it maintains cell viability while still eliciting sufficient wound‐responsive signaling, thereby optimizing transformation efficiency. Among the strains tested, ACCC10060—a strain that has been widely used for *A. rhizogenes*‐mediated transformation in *S. miltiorrhiza* (Li et al., 2017; Pan et al., 2023)—was selected as the optimal candidate due to its high transformation efficiency and its ability to induce morphologically normal roots suitable for downstream applications.

Similar to the CDB system, the present hypocotyl acupuncture method bypasses callus induction and subsequent aseptic manipulation, enabling direct *Agrobacterium* delivery into intact seedlings and thereby reducing reliance on labor‐intensive tissue culture procedures. The workflow is consequently simplified into seedling inoculation, selection of EGFP‐positive regenerants, and root harvesting, resulting in reduced procedural complexity. Despite sharing this general framework, the two systems differ in performance and applicability. The hypocotyl acupuncture method shows higher transformation efficiency in *S. miltiorrhiza* and utilizes a defined juvenile seedling stage that provides more standardized starting material and improved experimental reproducibility, compared with the more heterogeneous explants commonly used in CDB. Collectively, these features may support its applicability for efficient and scalable transformation in *S. miltiorrhiza*. These distinctions are summarized in Table S8.

Furthermore, as roots are the major medicinal tissues and primary sites for accumulation of pharmacologically active compounds in *S. miltiorrhiza* and many other medicinal plants, the *A. rhizogenes*‐mediated root transformation system provides a suitable platform for studying root metabolism and engineering bioactive compound production. In addition, because the hypocotyl acupuncture system does not rely on tissue culture regeneration and is compatible with standard CRISPR/Cas‐based vectors, it could potentially be extended to multiplex genome‐editing applications through appropriate vector design. Although multiplex editing was not directly evaluated in this study, the flexibility of this platform provides a foundation for future genetic improvement and metabolic engineering of medicinal plants.

Several limitations should be noted. First, although a duration of approximately 5 months remains acceptable for current applications, the root growth rate can be accelerated through optimized nutrition and environmental conditions. With such optimization, the 3.0 g threshold could be reached within 3 months, reducing the total protocol duration to approximately 4 months. Second, *S. miltiorrhiza* is an outcrossing species, and the seeds used herein represent a genetically heterogeneous pool of heterozygous genotypes; homozygous lines—to our knowledge—have not been established for this species. Formal evaluation of genotype‐dependent effects was therefore not feasible. Nevertheless, the transformation efficiency achieved across this genetically diverse material, together with the consistent results obtained from two regionally differentiated *S. miltiorrhiza* types and the high efficiencies observed across three phylogenetically distant species, suggests that the method is broadly applicable and not highly genotype‐restricted. Verification using isogenic lines, once available, would further strengthen this conclusion. Third, the present study focused on the establishment and optimization of the transformation and genome‐editing pipeline, and the analyses were therefore restricted to T0 regenerated plants. Consequently, key parameters including germline transmission, heritability of induced edits, and phenotypic segregation in T1 progeny remain to be assessed. These experiments are currently underway and will be essential to fully validate the system for stable genetic manipulation.

Overall, this study provides a simplified and scalable transformation platform for *S. miltiorrhiza*, substantially improving transformation efficiency while reducing technical complexity. This system offers a practical foundation for tissue culture‐independent genetic engineering and facilitates the application of advanced genome editing tools in medicinal plants.

## AUTHOR CONTRIBUTIONS

ZS: Conceptualize and direct the study, supervise the project, acquire funding, and review and revise the manuscript. XL: Design the methodology, supervise the project, and review and revise the manuscript. CW: Implement key experiments, conduct formal analysis, curate the data, and write the original draft. JL: Perform formal analysis, participate in the investigation, validate the results, and contribute to manuscript review and editing. CL: Carry out research and investigation, and execute experiments. CL: Assist with the execution of experimental procedures. JC: Verify the experimental results. JW: Provide resources and supervision. XM: Manage project administration and research coordination. All authors have read and approved the final version of the manuscript.

## Conflict of Interest

The authors declare no conflicts of interest.

## Supporting information


Supplementary Methods (S1–S8).

**Figure S1.** Characterization of *SmCPS1*‐edited transformed roots.
**Figure S2.** Influence of root fresh weight on sprouting in *S. miltiorrhiza*.
**Figure S3.** CRISPR/Cas9‐induced mutations in *SmCPS1*.
**Figure S4.** HPLC analysis of secondary metabolites in SmCPS1‐edited plants.
**Table S1‐1.** Mean germination rate and statistical grouping across different root biomass targets.
**Table S1‐2.** Raw data for the effect of root biomass target on germination rate.
**Table S2‐1.** Analysis of Variance (ANOVA) for the effects of Agrobacterium strain, Infection Solution, and OD value on transformation efficiency in *S. miltiorrhiza*.
**Table S2‐2.** Raw data of Agrobacterium‐mediated transformation efficiency (Positive Rate) used across all tested treatments in *S. miltiorrhiza*.
**Table S3.** Survival assessment of seedlings after acupuncture‐mediated infection.
**Table S4.** Positions and orientation of target sites within the gene sequence.
**Table S5.** In silico off‐target prediction within CDS regions for the four gRNAs, with gene annotation.
**Table S6.** Genotyping analysis of *SmCPS1*‐edited plants by Nanopore amplicon sequencing.
**Table S7.** Summary of transformation efficiency in various plant species.
**Table S8.** Comparison of transformation methods in *S. miltiorrhiza*.

**Data S1.** Raw nanopore sequencing data of SmCPS1.

## Data Availability

The raw sequence data reported in this paper have been deposited in the Genome Sequence Archive (Genomics, Proteomics & Bioinformatics 2021) in National Genomics Data Center (Nucleic Acids Res 2022), China National Center for Bioinformation/Beijing Institute of Genomics, Chinese Academy of Sciences (GSA: CRA029812) that are publicly accessible at https://ngdc.cncb.ac.cn/gsa.
