# Supplementary material for: Hypocotyl acupuncture: an efficient and practical protocol for transformation and genome editing in Salvia miltiorrhiza
Source: Plant J. 2026 Oct 7;128(1):e71103. doi: 10.1111/tpj.71103 (PMC13646370; doi:10.1111/tpj.71103)
Supplement: Supplementary file 1 [file TPJ-128-0-s003.docx]

**Supplementary Methods: Title:** Hypocotyl Acupuncture: An Efficient and Practical Protocol for Transformation and Genome Editing in *Salvia miltiorrhiza*

**S1. Plant Materials and Growth Conditions**

Seeds of *Salvia miltiorrhiza* were obtained from two sources: (i) commercial mixed-genotype populations purchased from the Anguo medicinal herb market (Hebei, China); and (ii) locally collected Baihua Danshen (white-flowered type) and Zihua Danshen (purple-flowered type) from Laiwu and Xintai, Shandong Province, respectively, harvested in the 2023 growing season. The Baihua and Zihua materials were used in supplementary experiments. Seeds of *Astragalus membranaceus*, *Isatis indigotica*, and *Platycodon* grandiflorum were also obtained from the Anguo market. All commercial seeds were used without separation by batch.To evaluate the protocol across diverse genetic backgrounds, we intentionally used mixed-genotype commercial seeds, which provide broader genetic diversity than a single inbred line. Consistent with the simplified, non-sterile protocol, no surface sterilization was performed. Seeds were germinated on double-layered moistened filter paper for approximately 10–15 days for *S. miltiorrhiza* (to ensure optimal hypocotyl diameter) and 7 days for *A. membranaceus* and *I. indigotica*. *P. grandiflorum* seeds were sown directly in soil and cultivated for approximately one month until reaching the juvenile stage suitable for transformation. All materials were maintained in a growth chamber at 25 °C under a 16-h light/8-h dark photoperiod.

**S2. Preparation of Infection Solutions**

Solution 1: Contained 10 mM MES (pH 5.6–5.8), 150 µM acetosyringone (AS), and 10 mM MgCl₂. This was used for a 3-hour pre-incubation of *Agrobacterium* cultures to enhance virulence.

Solution 2: Contained deionized water supplemented with 5% (w/v) sucrose and 0.02% (v/v) Silwet L-77.

Control: Sterile distilled water.

**S3. Vector Construction and *Agrobacterium* Preparation**

The binary vector pBI121-EGFP was utilized for initial protocol optimization. For CRISPR/Cas9 experiments, a modified pHSE401 vector, incorporating an *EGFP* expression cassette, was employed to facilitate fluorescence screening of transgenic events. Four gRNAs targeting a key flowering pathway gene (Table S4) were designed and integrated into the CRISPR/Cas9 backbone. PCR was performed using a high-fidelity DNA polymerase (Cat. No. AG12202; Accurate Biotechnology (Hunan) Co., Ltd., Changsha, China). All restriction endonucleases were purchased from Best Enzymes, Inc. (Lianyungang, China).

The constructs were introduced into *Agrobacterium rhizogenes* strains K599 (Strᴿ), Ar.Qual (Strᴿ), and ACCC10060 (Rifᴿ) via the freeze-thaw method using liquid nitrogen. Transformants were cultured at 28 °C in the following media: K599 and Ar.Qual were grown in TY medium (5 g/L tryptone, 3 g/L yeast extract, 10 mM CaCl₂) supplemented with 50 mg/L streptomycin and 50 mg/L kanamycin, while ACCC10060 was grown in YEB medium supplemented with 50 mg/L rifampicin and 50 mg/L kanamycin.

All strains were incubated until the OD₆₀₀ reached 0.8–1.2. Cells were harvested by centrifugation (5,000 rpm, 5 min), washed, and resuspended in the specified infection solution (Solution 1 or 2) to the required concentration for plant transformation.

**S4. Plant Transformation Procedure**

Transformatio*n* was executed via the hypocotyl needle-puncture method. Seedlings at the appropriate developmental stage (e.g., *S. miltiorrhiza*: approximately 3 cm in length with 2–4 true leaves or two fully expanded cotyledons) were selected. For each seedling, the *Agrobacterium* suspension was delivered into the juvenile hypocotyl via precise piercing and injection using a 1-mL syringe (<10 s per plant). Following injection, seedlings were immersed in the bacterial suspension for 1 h. This incubation period was utilized as a parallel processing stage to prepare or inoculate subsequent batches, ensuring a continuous, high-throughput workflow.

Post-infection, plants were transplanted into moist soil and initially maintained under controlled growth chamber conditions. Adventitious roots were typically induced and screened for *EGFP* fluorescence within 2–3 weeks. To facilitate rapid biomass accumulation and primary root system establishment, *EGFP*-positive individuals were subsequently transferred to a greenhouse under natural light conditions. Non-transgenic tissues were then excised from the developed root system to initiate shoot regeneration. The comprehensive process, from initial infection to the acquisition of fully regenerated transgenic plantlets, was achieved in approximately 5 months.

**S5. Molecular Detection and Analysis**

EGFP fluorescence in transgenic materials was detected using a LUYOR-3415RG dual-wavelength fluorescent protein excitation light source. Genomic DNA was extracted from fluorescent roots and regenerated leaves, and PCR amplification of the EGFP coding region was performed for molecular validation.

For NGS-based Mutation Analysis, PCR amplicons spanning the four gRNA target sites were sequenced. Raw data processing utilized a standard bioinformatics pipeline: fastp performed quality control and adapter trimming; clean reads were aligned to the reference gene sequence using BWA-MEM; and BCFtools conducted variant calling. The filtered variants were used to generate consensus FASTA sequences for individual alleles, which were subsequently analyzed via Multiple Sequence Alignment (MSA) using Jalview to assess specific mutation patterns. The raw sequence data reported in this paper have been deposited in the Genome Sequence Archive (GSA: CRA029812) and are publicly accessible at <https://ngdc.cncb.ac.cn/gsa>.

**S6. Statistical Analysis**

For both transformation and regeneration assays, each experimental group contained 20 seedlings. The number of replicate groups was increased in specific treatment combinations with low initial success rates to minimize experimental error and ensure statistical power. Statistical significance was assessed at a threshold of P < 0.05.

For transformation efficiency, 45 independent experimental groups (R1–R45) were established. A factorial analysis of variance (ANOVA) using Type III sums of squares was performed with the Anova function in the R package car (R version 4.2.1) to evaluate the main effects of Agrobacterium strain, infection solution, and OD₆₀₀ value.

Shoot regeneration was evaluated across root biomass targets (0.5–3.5 g). For each target, 3–6 biological replicate groups were used. Data were expressed as mean ± standard deviation (SD). One‑way ANOVA followed by Duncan’s multiple range test (α = 0.05) was used for post‑hoc comparisons.

All statistical analyses were performed in R, and graphical outputs were generated using R and Microsoft Excel 2019.

**S7. Off-target analysis**

Potential off-target sites for the four gRNA protospacers (19 bp, NGG PAM, sequences in Table S5) were searched against the *S. miltiorrhiza* reference genome using Cas-OFFinder (v2.4.1). Up to three mismatches were allowed. Only off-target loci located within CDS were retained for further consideration, as non-coding edits are generally considered non-deleterious.

For candidate off-target sites with 0–3 mismatches within CDS regions, PCR primers were designed to amplify ~300–500 bp fragments spanning each predicted site. Genomic DNA was extracted from 3–5 independent edited lines (confirmed for on-target editing) and wild-type controls. PCR products were subjected to Oxford Nanopore sequencing.

**S8. Extraction and HPLC analysis of tanshinone compounds**

Root samples were dried and ground into powder. Approximately 0.3 g of sample powder was extracted with 50 mL methanol by ultrasonic extraction for 30 min. The extracts were filtered through a 0.22 μm membrane filter before HPLC analysis.

HPLC analysis was performed using a reverse-phase C18 column with acetonitrile and aqueous solution as the mobile phases under gradient elution conditions. The flow rate was maintained at 1.0 mL/min, the column temperature was set at 20°C, the injection volume was 10 μL, and the detection wavelength was 270 nm.

The peak areas of tanshinone compounds were recorded and analyzed based on the corresponding retention times of authentic standards.
